# Herbal medicine and acupuncture relieved progressive bulbar palsy for more than 3 years: A case report

**DOI:** 10.1097/MD.0000000000031446

**Published:** 2022-11-11

**Authors:** Siyang Peng, Weiqian Chang, Yukun Tian, Yajing Yang, Shaohong Li, Jinxia Ni, Wenzeng Zhu

**Affiliations:** a Guang’anmen Hospital, China Academy of Chinese Medical Sciences, Xicheng District, Beijing, China; b Dongzhimen Hospital of Beijing University of Chinese Medicine, Dongcheng District, Beijing, China.

**Keywords:** acupuncture, case report, motor neuron disease (MND), progressive bulbar palsy (PBP), traditional Chinese medicine (TCM)

## Abstract

Progressive bulbar palsy (PBP) is a type of motor neuron disease (MND). The main symptoms include dysarthria, dysphagia, tongue muscle atrophy and fasciculations. This disease is generally severe and develops rapidly. Due to the lack of effective treatment, many patients with MND in China turn to traditional Chinese medicine treatment for help. We successfully relieved dysphagia and sialorrhea in a patient with PBP for 3 years with herbal medicine and acupuncture.

**Patient concerns:**

The patient was a 68-years-old woman with PBP and suffered from severe dysphagia and sialorrhea.

**Diagnoses:**

Progressive bulbar palsy.

**Interventions:**

Chinese herbal medicine and acupuncture.

**Outcomes:**

After 4 months of herbal medicine and acupuncture treatment, dysphagia and sialorrhea were relieved considerably. The patient’s condition has been stable for more than 3 years and continues to be treated with Chinese herbal medicine and acupuncture.

**Lessons:**

Our case suggests that alternative therapies such as herbal medicine and acupuncture may be effective in alleviating the symptoms of MND/PBP. However, standardized clinical studies are still required to verify the effectiveness and safety.

## 1. Introduction

Motor neuron disease (MND) is characterized by the degeneration of both upper and lower motor neurons, leading to muscle weakness and eventual paralysis.^[1]^ The progressive neurological deterioration involves the corticospinal tract, brainstem and anterior horn cells of the spinal cord.^[2]^ Death generally occurs within 2 to 4 years due to respiratory failure.^[3]^ The most common motor neuron disease is amyotrophic lateral sclerosis (ALS). Other forms include primary lateral sclerosis, progressive muscular atrophy, and progressive bulbar palsy (PBP).^[4]^ The initial symptom of MND is often extremity weakness; about 70% of patients present with this “limb-onset” disease. The remaining 25% present with dysarthria and dysphagia, and about 5% of patients present with trunk or respiratory symptoms at the onset.^[5]^ According to a systematic analysis of the global burden of MND in 2016, the worldwide all-age prevalence was 4.5 (4.1–5.0) per 1,00,000 people, and the all-age incidence was 0.78 per 1,00,000 person-years.^[6]^

The pathophysiology of MND remains unknown, which limits the development of disease-modifying therapies.^[7]^ The only 2 approved drugs for the treatment of MND are riluzole and edaravone.^[8,9]^ Riluzole can only prolong the median survival time by approximately 2 to 3 months in patients with ALS.^[9]^ It is still unclear whether edaravone therapy prolongs survival in the long term.^[10]^ Due to the lack of effective treatment, many patients with MND in China turn to traditional Chinese medicine (TCM) treatment.^[11]^ Studies have verified the effectiveness of acupuncture in improving swallowing ability after stroke.^[12,13]^ It has also been reported that Chinese herbal medicine and acupuncture may be an effective treatment for MND, relieving symptoms and improving quality of life.^[14,15]^ However, there are few studies that investigate the effectiveness and safety of traditional Chinese medicine in treating dysphagia and sialorrhea in patients with MND. Here, we report a case of successfully alleviating the symptoms of dysphagia and sialorrhea for a patient with PBP with Chinese herbal medicine and acupuncture for more than 3 years. The ethics committee of Guang’anmen Hospital, China Academy of Chinese Medical Sciences approved the study. The patient has provided informed consent for publication of this case and the writing of this case followed CARE guidelines.

## 2. Case presentation

### 2.1. Clinical presentation

**Figure 1.**
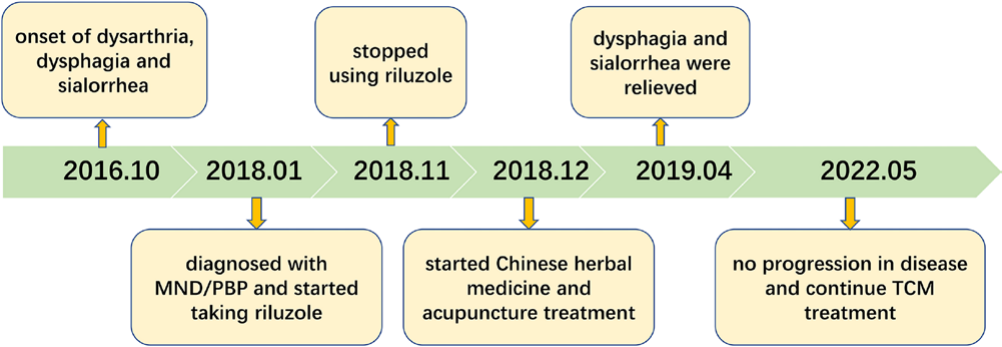
Timeline of clinical and procedural data.

The timeline with clinical and procedural data is shown in Figure 1.

The patient was a 68-year-old lady, who in October 2016, presented with dysarthria, dysphagia, and sialorrhea of unknown origin. There was no tongue numbness, abnormal taste, hoarseness, dyspnea, diplopia or blurred vision, limb weakness or numbness, dizziness, or loss of consciousness. The local hospital did not give a precise diagnosis, and provided treatments for cerebral infarction. After treatment with antiplatelets, circulation improvement and reducing her lipid, the symptoms did not improve and aggressively progressed. On January 9, 2018, the patient sought medical advice at Beijing Xuanwu Hospital, National Center for Neurological Disorders of China. Physical examination showed normal function of the advanced cortex, bilateral tongue muscle atrophy, fibrillation, normal pharyngeal reflex, uvula in the middle, and negative mandibular reflex. Muscle strength and muscular tension were normal, and the reflexes of the bilateral biceps brachii, triceps brachii, and radial membrane were hyperactive. The left palmomaxillary reflex was positive, the pathological signs of both lower limbs were negative, and the water-swallowing test result was grade 2. The mini-mental state examination score was 30. Supplementary examination showed no obvious abnormality in the blood, urine, stool, or cerebrospinal fluid. She underwent head and cervical MRI. The head MRI showed a right frontal subcortical punctate ischemic focus while the cervical MRI showed C4 to C5 and C5 to C6 intervertebral disc herniation. On electromyography images, the sensory conduction velocity of the double median nerve (finger I and finger III) had slowed and the amplitude of sensory conduction of the left median nerve (finger I) had decreased. Neuropsychological examination, carotid ultrasound, and intracranial artery ultrasound showed no obvious abnormality.

The patient was an elderly female with unclear onset and progressive aggravation of symptoms, mainly manifested as bulbar paralysis. The first consideration was neurodegenerative disease involving the bulbar for qualitative diagnosis, emphasizing PBP. The onset type of ALS was not excluded from the differential diagnosis. The patient had no evidence of involvement of the anterior horn of the spinal cord, such as limb muscle weakness, atrophy, or muscle fasciculation. The electromyography results were not that illuminating and the patient was diagnosed with MND/PBP. She began treatment with riluzole 50 mg twice per day to inhibit glutamate release. Mecobalamin 0.5 mg and vitamin B_1_ 10 mg 3 times per day were prescribed to improve nerve function. After taking riluzole for 10 months, the patient’s symptoms gradually worsened, and she stopped taking the drug.

On December 7, 2018, the patient came to our traditional Chinese medicine hospital for treatment. Symptoms at the time of her first visit were as follow: dysarthria, dysphagia, and excessive saliva, which required using a handkerchief. She had atrophy and fibrillation of tongue muscle, weakness of limbs, feeling of limb muscle fasciculation, and no apparent atrophy of limb muscles. She reported poor sleep quality, a weight loss of 5 kg due to poor nutrition in the past 6 months, constipation, normal urination, pink tongue and greasy coating, and deep and slow pulse.

### 2.2. Interventional procedure

According to TCM theory, we determined that she had flaccid disease and the syndrome of deficiency of spleen and kidney yang. The herbal medicine prescription was formulated to strengthen the spleen and kidney, supplement qi, and warm yang. Huangqi Shengji decoction was the main prescription of Chinese herbal medicine. The specific medication and dosage were as follows: milkvetch root 80 g, cassia twig 15 g, Chinese angelica 30 g, prepared rehmannia root 10 g, debark peony root 30 g, Sichuan lovage rhizome 10 g, suberect spatholobus stem 30 g, prepared common monkshood branched root 30 g, ephedra 15 g, alum processed pinellia 15 g, thunberbg fritillaria bulb 10 g, loquat leaf 30 g, inula flower 20 g, 2-toothed achyranthes root 15 g, manchurian wild ginger 10 g, spine date seed 30 g, tuber fleeceflower stem 30 g, liquorice root 10 g, golden thread 15 g, snakegourd fruit 30 g, largehead atractylodes rhizome 30 g, deer-horn glue 6 g, human placenta 3 g, nux vomica 0.3 g. The herbal medicines were decocted with water, about 200 ml each time, twice a day.

**Figure 2.**
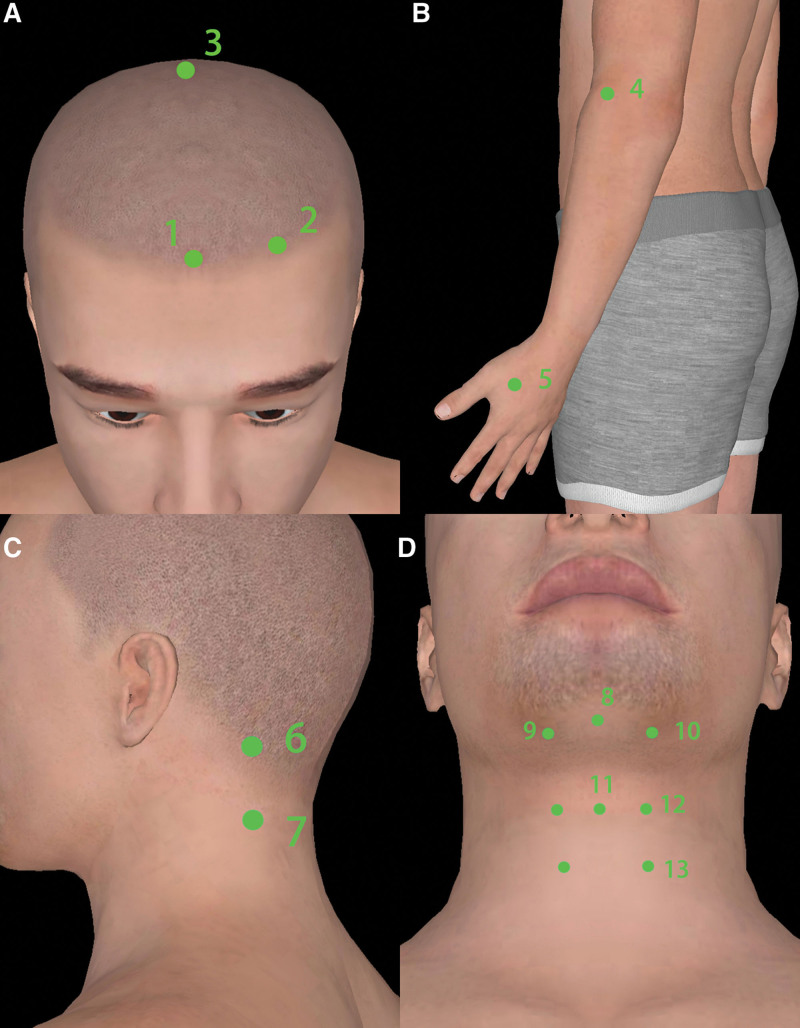
Positions of acupoints. (A) 1 Shenting (GV24), 2 Toulinqi (GB15), 3 Baihui (GV20). (B) 4 Hegu (LI4), 5 Quchi (LI11). (C) Fengchi (GB20), Gongxue (Extra). (D) 8 Lianquan (CV23), 9 Wai Yuye (Extra), 10 Wai Jinjin (Extra), 11 Zhiqiang (Extra), 12 Tunyan (Extra), 13 Fayin (Extra).

For acupuncture treatment, unilateral Lianquan (CV23) and Zhiqiang^[16]^ (Extra, between hyoid and the upper notch of thyroid cartilage), bilateral Fengchi (GB20), Hegu (LI4), Toulinqi (GB15), Shenting (GV24), Baihui (GV20), Quchi (LI11), Gongxue^[16]^ (Extra, 40 mm below Fengchi), Tunyan^[16]^ (Extra, between hyoid and prominentia laryngea, 5 mm next to the anterior median line), Fayin^[16]^ (Extra, 5 mm next to the median line under prominentia laryngea, between the thyroid cartilage and cricoid cartilage) and Wai Jinjin Yuye (Extra, 25 mm next to Lianqua, the left side is Wai Jinjin and the right side is Wai Yuye) were chosen. The positions of the acupoints are shown in Figure 2. We inserted 0.30 × 25 mm stainless steel, single-use, sterile needles 3 to 5 mm vertically at Tunyan, Fayin and Zhiqiang and 0.30 × 40 mm needles 30 to 35 mm toward the root of the tongue at Wai Jinjin Yuye and CV23. After insertion, gentle and even manipulations involving twirling and rotsating at a frequency of 180/minute were performed to attain deqi^[17]^ (a sensation of soreness, aching, swelling, numbness, or heaviness) at these acupoints. After twirling for 15 seconds, the needles were pulled out. 0.30 × 40 mm needles were inserted to a depth of 20 to 30 mm at Gongxue, GB20, LI4, GB15, GV24, GV20, and LI11 and were kept for 30 minutes. The GB20 was connected with electroacupuncture. A continuous wave was given; the frequency was 2 Hz, and the current intensity was 2 mA. The treatment frequency was once every other day, 3 times a week.

### 2.3. Follow-up and patient perspective

**Figure 3.**
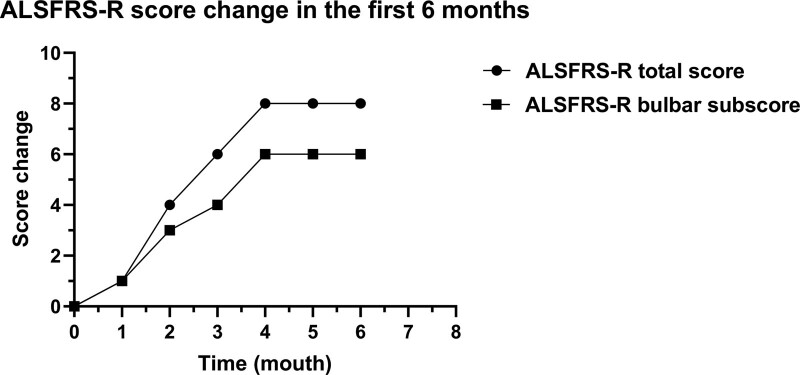
ALSFRS-R total score and bulbar subscore. ALSFRS-R = amyotrophic lateral sclerosis functional rating scale revised.

On January 18, 2019, 1 month after the combined acupuncture and herbal medicine treatment, the patient’s saliva decreased slightly, fatigue improved, and appetite increased. The other symptoms were the same as before. We slightly adjusted the herbal medicine prescription and continued the acupuncture treatment. On March 12, 2019, after 3 months of treatment, saliva decreased significantly, and the frequency of limb muscle fasciculation decreased. On April 2, 2019, after 4 months of treatment, she had less saliva, dysphagia was relieved considerably, and she had no problem eating. The strength of limbs was enhanced, and sleep condition was also improved. The change in amyotrophic lateral sclerosis functional rating scale revised score in the first 6 months of treatment is depicted in Figure 3. In a later follow-up, the patient’s symptoms were stable. Her herbal medicine prescription was changed slightly according to the syndromes once or twice a month, and acupuncture treatment was performed two to 3 times a week. During treatment, no abnormality was found in liver and kidney function testing. The patient’s condition has been stable for more than 3 years and continues to be treated with Chinese herbal medicine and acupuncture in our clinic.

## 3. Discussion

PBP is a form of MND, which is less common than ALS.^[4]^ Among patients with PBP, 87% progress to definite ALS.^[18]^ Epidemiological statistics show that PBP accounts for 4.1% of MND in China.^[19]^ The onset age of PBP is generally late, mainly after 40 or 50 years of age. The main symptoms include dysarthria, dysphagia, tongue muscle atrophy, and fasciculations.^[2]^ This type of disease is generally severe and develops rapidly,^[20]^ and most patients die of respiratory muscle paralysis or lung infection within 1 to 2 years.^[21]^ The mechanisms underlying neurodegeneration in MND remain incompletely understood. Many cellular and molecular processes have been implicated,^[22]^ including toxic protein aggregation, mitochondrial dysfunction, excitotoxicity, hypermetabolism, oxidative stress, and inflammation. The only 2 approved drugs for the treatment of MND are riluzole and edaravone.^[8,9]^ A 50 mg dose, twice a day for 18 months, of riluzole, a glutamatergic neurotransmission inhibitor, can delay the course of the disease and prolong the median survival time by about 2 to 3 months in patients with ALS.^[9]^ Edaravone, a free-radical scavenger of peroxyl radicals, showed efficacy in a small subset of people with MND in maintaining function and quality of life in the early stage.^[10,23]^ It is still unclear whether edaravone therapy prolongs survival in the long term.^[10]^ Although promising outcomes were obtained in preclinical studies, numerous compounds investigated failed in human clinical trials,^[24,25]^ and there is no available treatment to stop or reverse the progressive course of MND.^[26]^ Symptomatic treatments include the treatment of cramps,^[27]^ pain,^[28]^ spasticity,^[29]^ noninvasive ventilation for supporting respiratory function,^[30]^ and enteral tube feeding to support nutrition deficiencies.^[31]^

The main complaint of our patient was progressive dysphagia and sialorrhea. For dysphagia, feeding tube placement and percutaneous endoscopic gastrostomy (PEG) may be necessary if the patient has poor nutrition and loses weight. For sialorrhea, botulinum toxin type-B injections to parotid and submandibular glands are mostly effective in the short term (up to 4 weeks). However, there is probably no benefit beyond this time after a single injection.^[32]^ Anticholinergic medications (amitriptyline and glycopyrronium bromide) are often used for treating sialorrhea, but there is not enough evidence proving the efficacy of these drugs in MND.^[31,33]^ The patient turned to TCM for symptom relief. After 4 months of herbal medicine combined with acupuncture treatment, the dysphagia and sialorrhea were significantly reduced, and her quality of life improved markedly. She avoided a PEG and feeding tube insertion.^[32]^ For this patient, almost 6 years have passed since the onset of symptoms, and the treatment has been maintained for more than 3 years. The disease has not deteriorated or progressed to ALS, and a relatively good treatment effect has been achieved.

Under TCM theory, we believe that the patient belongs to the syndrome of deficiency of spleen and kidney yang.^[34]^ Therefore, various herbal medicines are used to tonify the spleen and kidney, warm yang, and replenish qi. TCM is a complementary and alternative treatment for MND, especially in China, and includes herbal medicine, massage, acupuncture, moxibustion, and other methods, among which herbal medicine and acupuncture are most commonly used.^[11,35]^ To alleviate the symptoms, 90% of patients take Chinese herbal production in Shanghai, China.^[36]^ In animal models, TCM improved motor function and extended survival duration by inhibiting inflammation. Herbal medicines have been testified to prolong survival duration and relieve symptoms for patients with MND in some case reports and clinical studies.^[37,38]^ However, the credibility of these findings is limited by the non-RCT design, unverified outcome measures, a small sample size, or short follow-up. Thus, these reports cannot provide evidence-based support for the clinical use of TCM in the treatment of MND. A prospective registry study has been conducted in China to clarify whether TCM is an appropriate therapy for patients with MND (CARE-TCM).^[35]^ This study will help identify common diagnostic approaches and treatment modalities among Chinese patients with ALS receiving TCM treatment, enabling the establishment of strategies for treatment based on evidence-based medicine. To promote the application of herbal medicine as an alternative therapy in the treatment of MND, animal experiments that explain the pharmacology and toxicology and large-scale and rigorously designed high-quality clinical studies should be performed.^[14]^

Acupuncture is a type of complementary and alternative medicine that has been widely used in China, Korea, and Japan for centuries.^[11]^ Experiments on animals suggest that electroacupuncture treatment can help increase anti-inflammation activity in the central nervous system and respiratory system of animals with MND.^[39]^ Unfortunately, the number of clinical studies on acupuncture for the treatment of MND is minimal. In our case, we used neck acupuncture, which is often used for aphasia or dysphagia caused by bulbar paralysis after stroke, to solve the problem of dysphagia and sialorrhea. Its effectiveness and safety have been confirmed by many clinical studies.^[40]^ Commonly used acupoints include Lianquan, Wai Jinjin Yuye, Renying, Tiantu, and Fengchi.

We do acknowledge that it is hard to determine whether the combined treatment is superior to herbal medicine or acupuncture, when used alone, in alleviating symptoms and improving quality of life of patients with MND/PBP. When herbal medicine and acupuncture are used simultaneously, the efficacy of the 2 therapies cannot be distinguished, either. In TCM hospitals, due to the rapid progression of the disease and difficulty in treatment, patients with MND are managed with multiple TCM therapies. Each therapy has its own indications and limitations. Acupuncture is good at improving dysphagia while herbal medicine is good at improving some of the associated symptoms. For acupuncture treatment, patients have to come to the hospital 3 times a week, whereas herbal medicine can be taken at home to ensure the continuity of treatment when patients are unable to visit the hospital.

Our case report suggests that acupuncture combined with Huangqi Shengji decoction may alleviate dysphagia and salivation in patients with PBP. When faced with patients with MND/PBP with dysphagia and salivation symptoms in clinical practice, TCM doctors or acupuncturists can use this combined treatment and observe the therapeutic effect. Due to the low incidence rate and the complexity of TCM interventions, it is difficult to conduct standardized randomized controlled trials to investigate the efficacy and safety of this combined treatment. However, case-control, prospective cohort, or observational studies can be conducted to observe the therapeutic effects. After the preliminary evaluation of the efficacy of the combined treatment, we can proceed to interventional studies.

## 4. Conclusion

This paper reports a case of Chinese herbal medicine combined with acupuncture in the treatment of MND/PBP that successfully alleviated dysphagia and sialorrhea. Our report suggests that alternative therapies, such as herbal medicine and acupuncture, may effectively reduce the symptoms of MND/PBP. However, standardized clinical studies are still required to verify the effectiveness and safety of this treatment.

## Author contributions

**Conceptualization:** Yajing Yang, Jinxia Ni.

**Resources:** Weiqian Chang, Yukun Tian.

**Visualization:** Shaohong Li.

**Writing – original draft:** Wenzeng Zhu.

**Writing – review & editing:** Siyang Peng.
